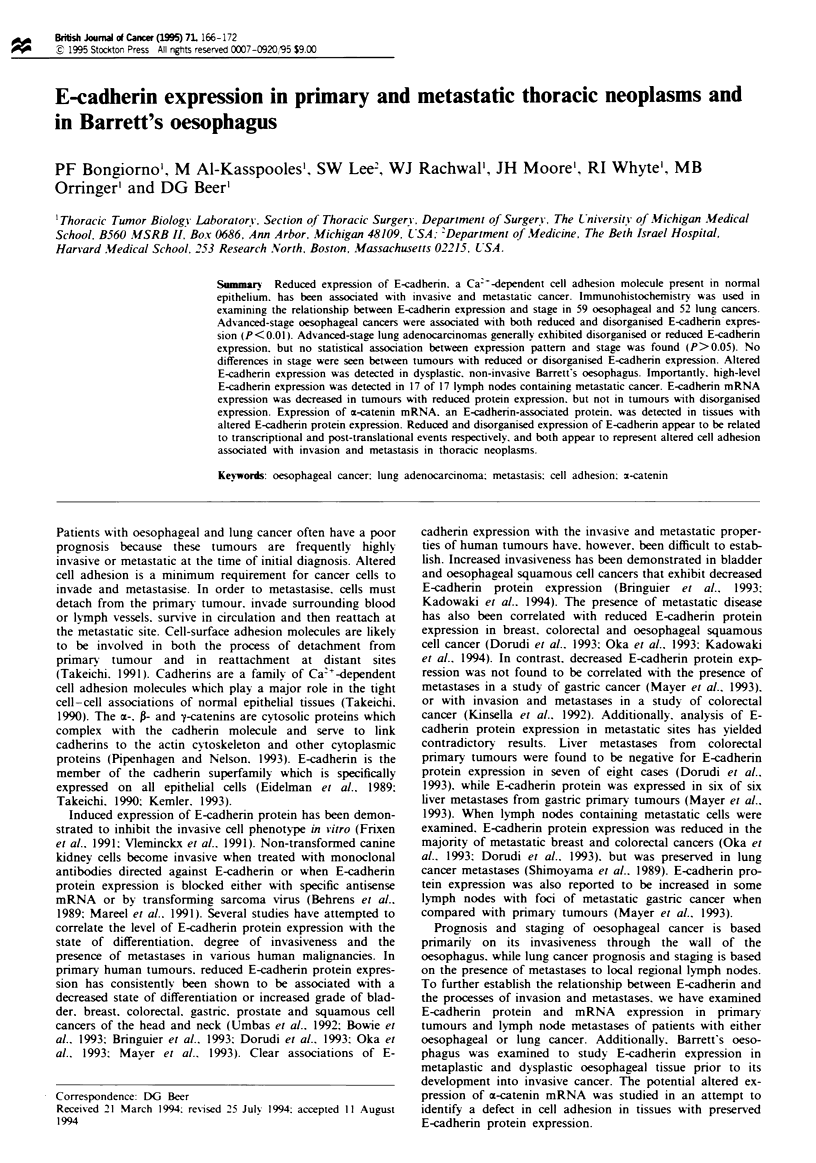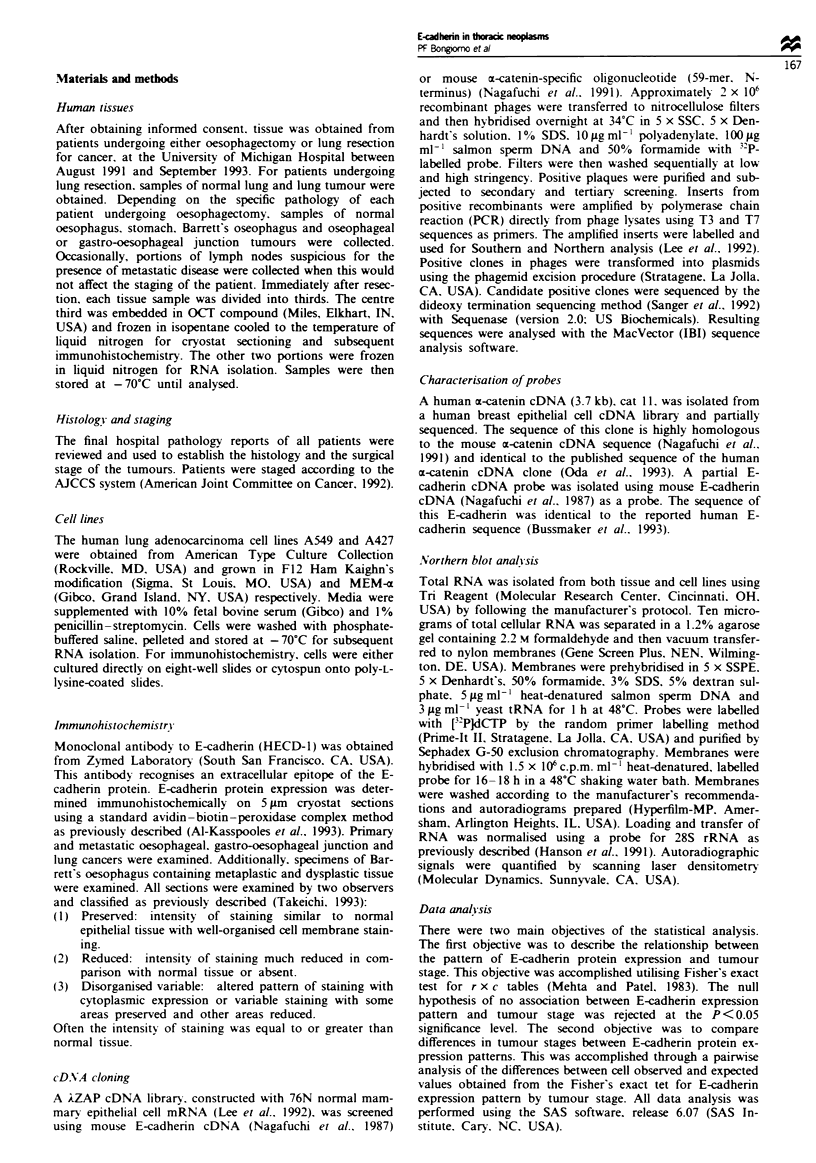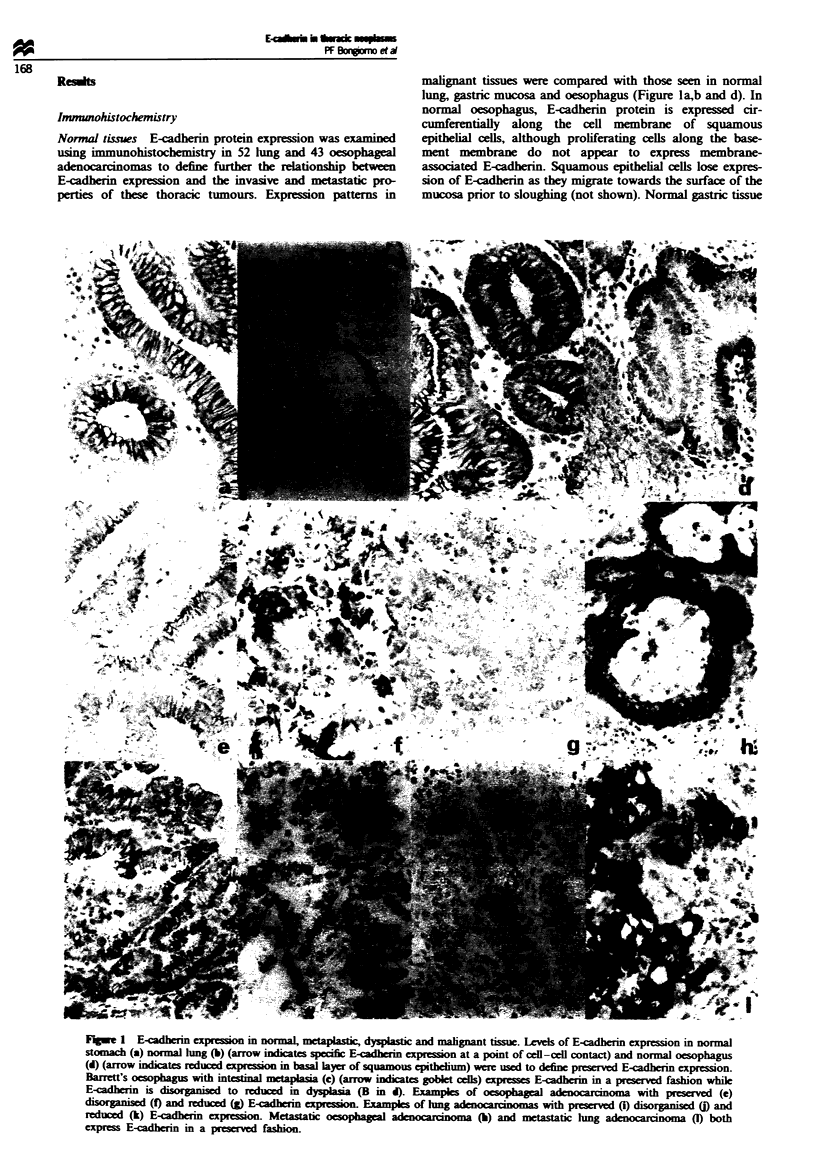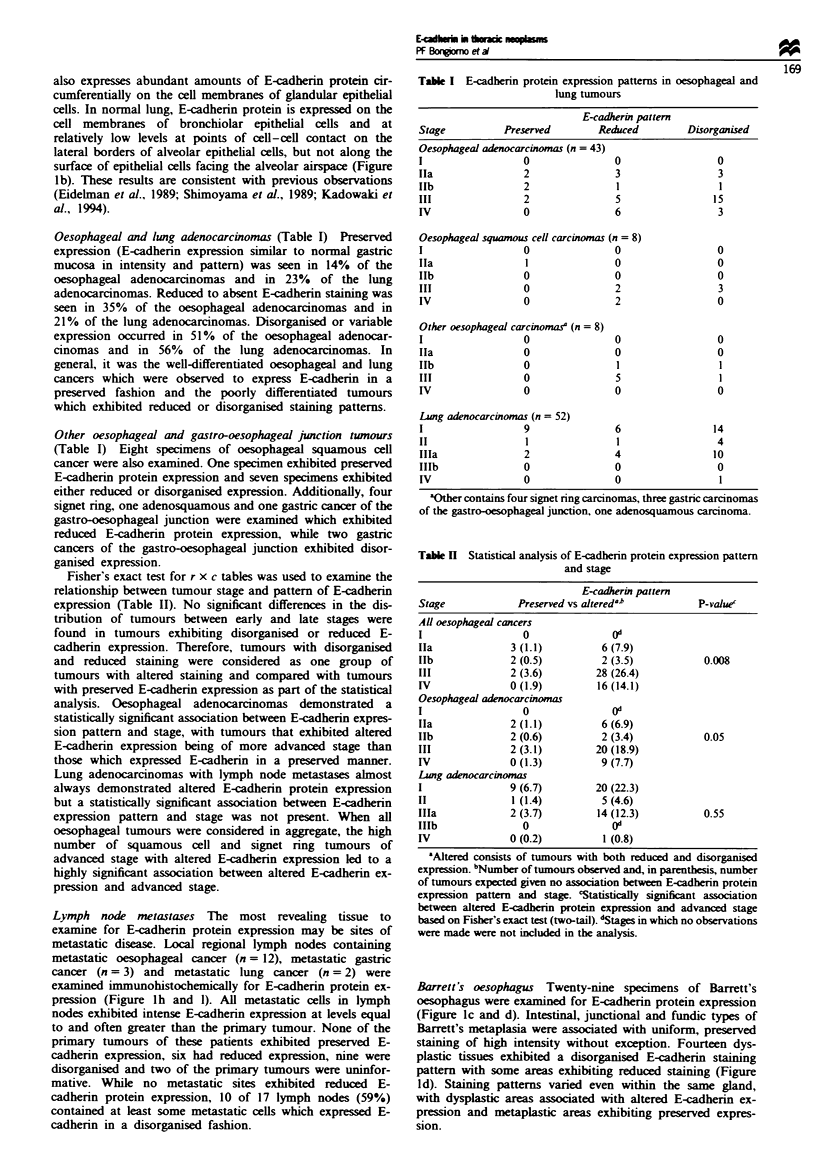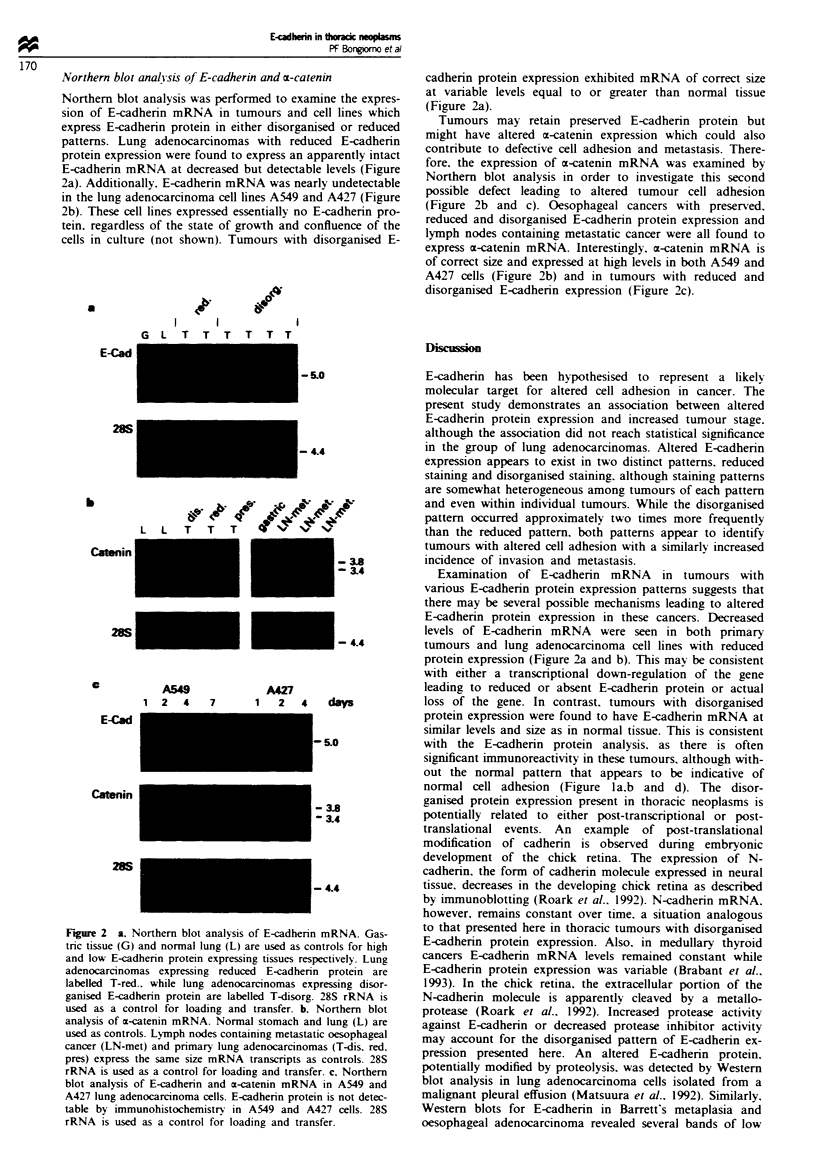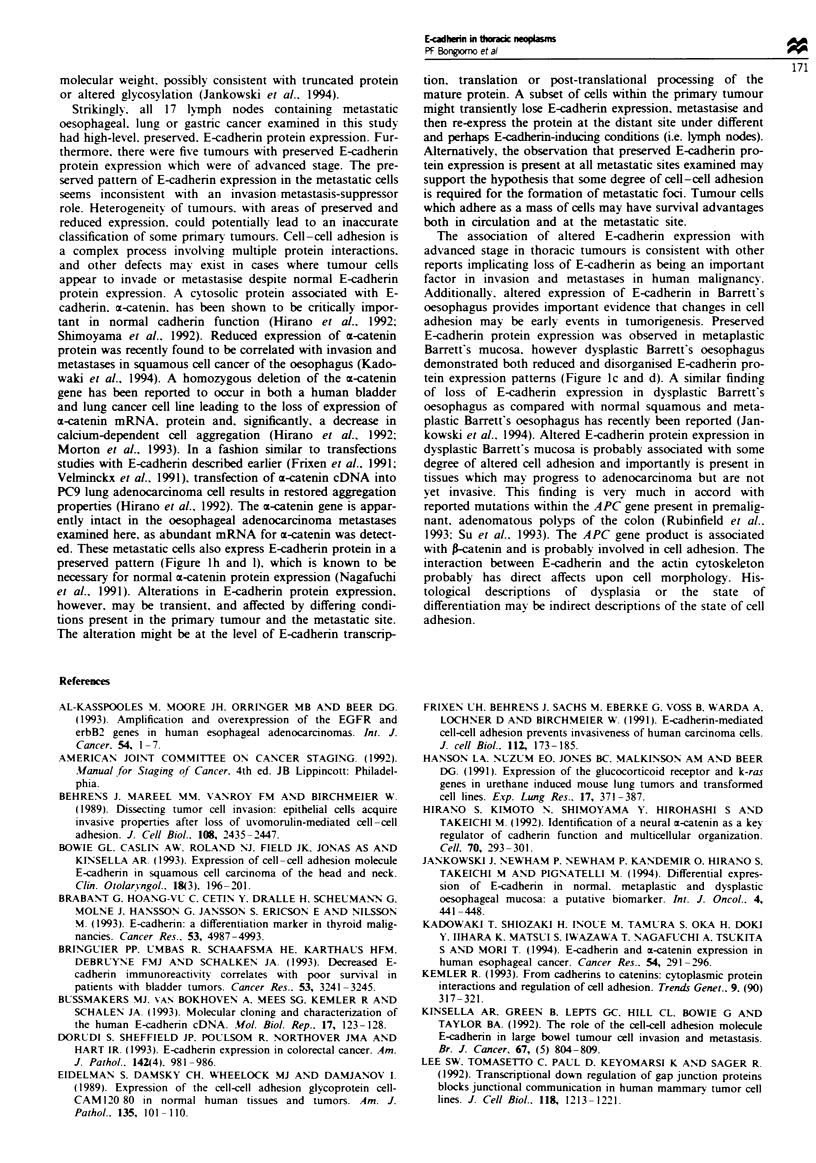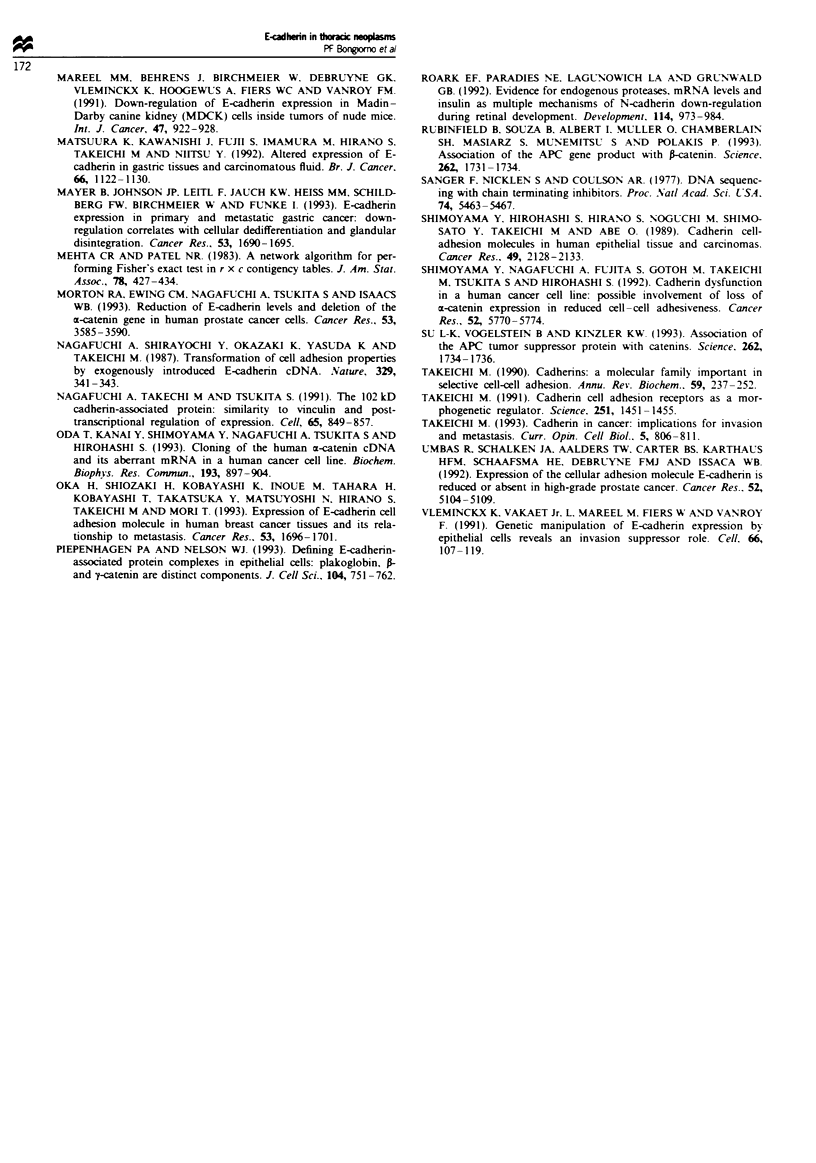# E-cadherin expression in primary and metastatic thoracic neoplasms and in Barrett's oesophagus.

**DOI:** 10.1038/bjc.1995.34

**Published:** 1995-01

**Authors:** P. F. Bongiorno, M. al-Kasspooles, S. W. Lee, W. J. Rachwal, J. H. Moore, R. I. Whyte, M. B. Orringer, D. G. Beer

**Affiliations:** Department of Surgery, University of Michigan Medical School, Ann Arbor 48109.

## Abstract

**Images:**


British Journal of Cancer (1995) 71, 166-172

© 1995 Stockton Press All rights reserved 0007-0920/95 $9.00

### E-cadherin expression in primary and metastatic thoracic neoplasms and in Barrett's oesophagus

PF Bongiorno[1], M Al-Kasspooles[1], SW Lee[2], WJ Rachwal[1], JH Moore[1], RI Whyte[1], MB Orringer[1] and DG Beer[1]

[1]*Thoracic Tumor Biology Laboratory, Section of Thoracic Surgery, Department of Surgery, The University of Michigan Medical School, B560 MSRB II, Box 0686, Ann Arbor, Michigan 48109, USA;* [2]*Department of Medicine, The Beth Israel Hospital, Harvard Medical School, 253 Research North, Boston, Massachusetts 02215, USA.*

**Summary** Reduced expression of E-cadherin, a $Ca^{2+}$-dependent cell adhesion molecule present in normal epithelium, has been associated with invasive and metastatic cancer. Immunohistochemistry was used in examining the relationship between E-cadherin expression and stage in 59 oesophageal and 52 lung cancers. Advanced-stage oesophageal cancers were associated with both reduced and disorganised E-cadherin expression ($P<0.01$). Advanced-stage lung adenocarcinomas generally exhibited disorganised or reduced E-cadherin expression, but no statistical association between expression pattern and stage was found ($P>0.05$). No differences in stage were seen between tumours with reduced or disorganised E-cadherin expression. Altered E-cadherin expression was detected in dysplastic, non-invasive Barrett's oesophagus. Importantly, high-level E-cadherin expression was detected in 17 of 17 lymph nodes containing metastatic cancer. E-cadherin mRNA expression was decreased in tumours with reduced protein expression, but not in tumours with disorganised expression. Expression of α-catenin mRNA, an E-cadherin-associated protein, was detected in tissues with altered E-cadherin protein expression. Reduced and disorganised expression of E-cadherin appear to be related to transcriptional and post-translational events respectively, and both appear to represent altered cell adhesion associated with invasion and metastasis in thoracic neoplasms.

**Keywords**: oesophageal cancer; lung adenocarcinoma; metastasis; cell adhesion; α-catenin

Patients with oesophageal and lung cancer often have a poor prognosis because these tumours are frequently highly invasive or metastatic at the time of initial diagnosis. Altered cell adhesion is a minimum requirement for cancer cells to invade and metastasise. In order to metastasise, cells must detach from the primary tumour, invade surrounding blood or lymph vessels, survive in circulation and then reattach at the metastatic site. Cell-surface adhesion molecules are likely to be involved in both the process of detachment from primary tumour and in reattachment at distant sites (Takeichi, 1991). Cadherins are a family of $Ca^{2+}$-dependent cell adhesion molecules which play a major role in the tight cell-cell associations of normal epithelial tissues (Takeichi, 1990). The α-, β- and γ-catenins are cytosolic proteins which complex with the cadherin molecule and serve to link cadherins to the actin cytoskeleton and other cytoplasmic proteins (Pipenhagen and Nelson, 1993). E-cadherin is the member of the cadherin superfamily which is specifically expressed on all epithelial cells (Eidelman *et al.*, 1989; Takeichi, 1990; Kemler, 1993).

Induced expression of E-cadherin protein has been demonstrated to inhibit the invasive cell phenotype *in vitro* (Frixen *et al.*, 1991; Vleminckx *et al.*, 1991). Non-transformed canine kidney cells become invasive when treated with monoclonal antibodies directed against E-cadherin or when E-cadherin protein expression is blocked either with specific antisense mRNA or by transforming sarcoma virus (Behrens *et al.*, 1989; Mareel *et al.*, 1991). Several studies have attempted to correlate the level of E-cadherin protein expression with the state of differentiation, degree of invasiveness and the presence of metastases in various human malignancies. In primary human tumours, reduced E-cadherin protein expression has consistently been shown to be associated with a decreased state of differentiation or increased grade of bladder, breast, colorectal, gastric, prostate and squamous cell cancers of the head and neck (Umbas *et al.*, 1992; Bowie *et al.*, 1993; Bringuier *et al.*, 1993; Dorudi *et al.*, 1993; Oka *et al.*, 1993; Mayer *et al.*, 1993). Clear associations of E-cadherin expression with the invasive and metastatic properties of human tumours have, however, been difficult to establish. Increased invasiveness has been demonstrated in bladder and oesophageal squamous cell cancers that exhibit decreased E-cadherin protein expression (Bringuier *et al.*, 1993; Kadowaki *et al.*, 1994). The presence of metastatic disease has also been correlated with reduced E-cadherin protein expression in breast, colorectal and oesophageal squamous cell cancer (Dorudi *et al.*, 1993; Oka *et al.*, 1993; Kadowaki *et al.*, 1994). In contrast, decreased E-cadherin protein expression was not found to be correlated with the presence of metastases in a study of gastric cancer (Mayer *et al.*, 1993), or with invasion and metastases in a study of colorectal cancer (Kinsella *et al.*, 1992). Additionally, analysis of E-cadherin protein expression in metastatic sites has yielded contradictory results. Liver metastases from colorectal primary tumours were found to be negative for E-cadherin protein expression in seven of eight cases (Dorudi *et al.*, 1993), while E-cadherin protein was expressed in six of six liver metastases from gastric primary tumours (Mayer *et al.*, 1993). When lymph nodes containing metastatic cells were examined, E-cadherin protein expression was reduced in the majority of metastatic breast and colorectal cancers (Oka *et al.*, 1993; Dorudi *et al.*, 1993), but was preserved in lung cancer metastases (Shimoyama *et al.*, 1989). E-cadherin protein expression was also reported to be increased in some lymph nodes with foci of metastatic gastric cancer when compared with primary tumours (Mayer *et al.*, 1993).

Prognosis and staging of oesophageal cancer is based primarily on its invasiveness through the wall of the oesophagus, while lung cancer prognosis and staging is based on the presence of metastases to local regional lymph nodes. To further establish the relationship between E-cadherin and the processes of invasion and metastases, we have examined E-cadherin protein and mRNA expression in primary tumours and lymph node metastases of patients with either oesophageal or lung cancer. Additionally, Barrett's oesophagus was examined to study E-cadherin expression in metaplastic and dysplastic oesophageal tissue prior to its development into invasive cancer. The potential altered expression of α-catenin mRNA was studied in an attempt to identify a defect in cell adhesion in tissues with preserved E-cadherin protein expression.

Correspondence: DG Beer
Received 21 March 1994; revised 25 July 1994; accepted 11 August 1994

### Materials and methods

### *Human tissues*

After obtaining informed consent, tissue was obtained from patients undergoing either oesophagectomy or lung resection for cancer, at the University of Michigan Hospital between August 1991 and September 1993. For patients undergoing lung resection, samples of normal lung and lung tumour were obtained. Depending on the specific pathology of each patient undergoing oesophagectomy, samples of normal oesophagus, stomach, Barrett's oseophagus and oseophageal or gastro-oesophageal junction tumours were collected. Occasionally, portions of lymph nodes suspicious for the presence of metastatic disease were collected when this would not affect the staging of the patient. Immediately after resection, each tissue sample was divided into thirds. The centre third was embedded in OCT compound (Miles, Elkhart, IN, USA) and frozen in isopentane cooled to the temperature of liquid nitrogen for cryostat sectioning and subsequent immunohistochemistry. The other two portions were frozen in liquid nitrogen for RNA isolation. Samples were then stored at −70°C until analysed.

### *Histology and staging*

The final hospital pathology reports of all patients were reviewed and used to establish the histology and the surgical stage of the tumours. Patients were staged according to the AJCCS system (American Joint Committee on Cancer, 1992).

### *Cell lines*

The human lung adenocarcinoma cell lines A549 and A427 were obtained from American Type Culture Collection (Rockville, MD, USA) and grown in F12 Ham Kaighn's modification (Sigma, St Louis, MO, USA) and MEM-α (Gibco, Grand Island, NY, USA) respectively. Media were supplemented with 10% fetal bovine serum (Gibco) and 1% penicillin–streptomycin. Cells were washed with phosphate-buffered saline, pelleted and stored at −70°C for subsequent RNA isolation. For immunohistochemistry, cells were either cultured directly on eight-well slides or cytospun onto poly-L-lysine-coated slides.

### *Immunohistochemistry*

Monoclonal antibody to E-cadherin (HECD-1) was obtained from Zymed Laboratory (South San Francisco, CA, USA). This antibody recognises an extracellular epitope of the E-cadherin protein. E-cadherin protein expression was determined immunohistochemically on 5 μm cryostat sections using a standard avidin–biotin–peroxidase complex method as previously described (Al-Kasspooles *et al.*, 1993). Primary and metastatic oesophageal, gastro-oesophageal junction and lung cancers were examined. Additionally, specimens of Barrett's oesophagus containing metaplastic and dysplastic tissue were examined. All sections were examined by two observers and classified as previously described (Takeichi, 1993):

(1) Preserved: intensity of staining similar to normal epithelial tissue with well-organised cell membrane staining.
(2) Reduced: intensity of staining much reduced in comparison with normal tissue or absent.
(3) Disorganised variable: altered pattern of staining with cytoplasmic expression or variable staining with some areas preserved and other areas reduced.

Often the intensity of staining was equal to or greater than normal tissue.

### *cDNA cloning*

A λZAP cDNA library, constructed with 76N normal mammary epithelial cell mRNA (Lee *et al.*, 1992), was screened using mouse E-cadherin cDNA (Nagafuchi *et al.*, 1987) or mouse α-catenin-specific oligonucleotide (59-mer, N-terminus) (Nagafuchi *et al.*, 1991). Approximately $2 \times 10^6$ recombinant phages were transferred to nitrocellulose filters and then hybridised overnight at 34°C in 5 × SSC, 5 × Denhardt's solution, 1% SDS, 10 μg ml$^{-1}$ polyadenylate, 100 μg ml$^{-1}$ salmon sperm DNA and 50% formamide with $^{32}$P-labelled probe. Filters were then washed sequentially at low and high stringency. Positive plaques were purified and subjected to secondary and tertiary screening. Inserts from positive recombinants were amplified by polymerase chain reaction (PCR) directly from phage lysates using T3 and T7 sequences as primers. The amplified inserts were labelled and used for Southern and Northern analysis (Lee *et al.*, 1992). Positive clones in phages were transformed into plasmids using the phagemid excision procedure (Stratagene, La Jolla, CA, USA). Candidate positive clones were sequenced by the dideoxy termination sequencing method (Sanger *et al.*, 1992) with Sequenase (version 2.0; US Biochemicals). Resulting sequences were analysed with the MacVector (IBI) sequence analysis software.

### *Characterisation of probes*

A human α-catenin cDNA (3.7 kb), cat 11, was isolated from a human breast epithelial cell cDNA library and partially sequenced. The sequence of this clone is highly homologous to the mouse α-catenin cDNA sequence (Nagafuchi *et al.*, 1991) and identical to the published sequence of the human α-catenin cDNA clone (Oda *et al.*, 1993). A partial E-cadherin cDNA probe was isolated using mouse E-cadherin cDNA (Nagafuchi *et al.*, 1987) as a probe. The sequence of this E-cadherin was identical to the reported human E-cadherin sequence (Bussmaker *et al.*, 1993).

### *Northern blot analysis*

Total RNA was isolated from both tissue and cell lines using Tri Reagent (Molecular Research Center, Cincinnati, OH, USA) by following the manufacturer's protocol. Ten micrograms of total cellular RNA was separated in a 1.2% agarose gel containing 2.2 M formaldehyde and then vacuum transferred to nylon membranes (Gene Screen Plus, NEN, Wilmington, DE, USA). Membranes were prehybridised in 5 × SSPE, 5 × Denhardt's, 50% formamide, 3% SDS, 5% dextran sulphate, 5 μg ml$^{-1}$ heat-denatured salmon sperm DNA and 3 μg ml$^{-1}$ yeast tRNA for 1 h at 48°C. Probes were labelled with [$^{32}$P]dCTP by the random primer labelling method (Prime-It II, Stratagene, La Jolla, CA, USA) and purified by Sephadex G-50 exclusion chromatography. Membranes were hybridised with $1.5 \times 10^6$ c.p.m. ml$^{-1}$ heat-denatured, labelled probe for 16–18 h in a 48°C shaking water bath. Membranes were washed according to the manufacturer's recommendations and autoradiograms prepared (Hyperfilm-MP, Amersham, Arlington Heights, IL, USA). Loading and transfer of RNA was normalised using a probe for 28S rRNA as previously described (Hanson *et al.*, 1991). Autoradiographic signals were quantified by scanning laser densitometry (Molecular Dynamics, Sunnyvale, CA, USA).

### *Data analysis*

There were two main objectives of the statistical analysis. The first objective was to describe the relationship between the pattern of E-cadherin protein expression and tumour stage. This objective was accomplished utilising Fisher's exact test for $r \times c$ tables (Mehta and Patel, 1983). The null hypothesis of no association between E-cadherin expression pattern and tumour stage was rejected at the $P<0.05$ significance level. The second objective was to compare differences in tumour stages between E-cadherin protein expression patterns. This was accomplished through a pairwise analysis of the differences between cell observed and expected values obtained from the Fisher's exact tet for E-cadherin expression pattern by tumour stage. All data analysis was performed using the SAS software, release 6.07 (SAS Institute, Cary, NC, USA).

### Results

### *Immunohistochemistry*

*Normal tissues* E-cadherin protein expression was examined using immunohistochemistry in 52 lung and 43 oesophageal adenocarcinomas to define further the relationship between E-cadherin expression and the invasive and metastatic properties of these thoracic tumours. Expression patterns in malignant tissues were compared with those seen in normal lung, gastric mucosa and oesophagus (Figure 1a,b and d). In normal oesophagus, E-cadherin protein is expressed circumferentially along the cell membrane of squamous epithelial cells, although proliferating cells along the basement membrane do not appear to express membrane-associated E-cadherin. Squamous epithelial cells lose expression of E-cadherin as they migrate towards the surface of the mucosa prior to sloughing (not shown). Normal gastric tissue

**Figure 1** E-cadherin expression in normal, metaplastic, dysplastic and malignant tissue. Levels of E-cadherin expression in normal stomach (**a**) normal lung (**b**) (arrow indicates specific E-cadherin expression at a point of cell–cell contact) and normal oesophagus (**d**) (arrow indicates reduced expression in basal layer of squamous epithelium) were used to define preserved E-cadherin expression. Barrett's oesophagus with intestinal metaplasia (**c**) (arrow indicates goblet cells) expresses E-cadherin in a preserved fashion while E-cadherin is disorganised to reduced in dysplasia (B in **d**). Examples of oesophageal adenocarcinoma with preserved (**e**) disorganised (**f**) and reduced (**g**) E-cadherin expression. Examples of lung adenocarcinomas with preserved (**i**) disorganised (**j**) and reduced (**k**) E-cadherin expression. Metastatic oesophageal adenocarcinoma (**h**) and metastatic lung adenocarcinoma (**l**) both express E-cadherin in a preserved fashion.

also expresses abundant amounts of E-cadherin protein circumferentially on the cell membranes of glandular epithelial cells. In normal lung, E-cadherin protein is expressed on the cell membranes of bronchiolar epithelial cells and at relatively low levels at points of cell–cell contact on the lateral borders of alveolar epithelial cells, but not along the surface of epithelial cells facing the alveolar airspace (Figure 1b). These results are consistent with previous observations (Eidelman *et al.*, 1989; Shimoyama *et al.*, 1989; Kadowaki *et al.*, 1994).

*Oesophageal and lung adenocarcinomas* (Table I) Preserved expression (E-cadherin expression similar to normal gastric mucosa in intensity and pattern) was seen in 14% of the oesophageal adenocarcinomas and in 23% of the lung adenocarcinomas. Reduced to absent E-cadherin staining was seen in 35% of the oesophageal adenocarcinomas and in 21% of the lung adenocarcinomas. Disorganised or variable expression occurred in 51% of the oesophageal adenocarcinomas and in 56% of the lung adenocarcinomas. In general, it was the well-differentiated oesophageal and lung cancers which were observed to express E-cadherin in a preserved fashion and the poorly differentiated tumours which exhibited reduced or disorganised staining patterns.

*Other oesophageal and gastro-oesophageal junction tumours* (Table I) Eight specimens of oesophageal squamous cell cancer were also examined. One specimen exhibited preserved E-cadherin protein expression and seven specimens exhibited either reduced or disorganised expression. Additionally, four signet ring, one adenosquamous and one gastric cancer of the gastro-oesophageal junction were examined which exhibited reduced E-cadherin protein expression, while two gastric cancers of the gastro-oesophageal junction exhibited disorganised expression.

Fisher's exact test for $r \times c$ tables was used to examine the relationship between tumour stage and pattern of E-cadherin expression (Table II). No significant differences in the distribution of tumours between early and late stages were found in tumours exhibiting disorganised or reduced E-cadherin expression. Therefore, tumours with disorganised and reduced staining were considered as one group of tumours with altered staining and compared with tumours with preserved E-cadherin expression as part of the statistical analysis. Oesophageal adenocarcinomas demonstrated a statistically significant association between E-cadherin expression pattern and stage, with tumours that exhibited altered E-cadherin expression being of more advanced stage than those which expressed E-cadherin in a preserved manner. Lung adenocarcinomas with lymph node metastases almost always demonstrated altered E-cadherin protein expression but a statistically significant association between E-cadherin expression pattern and stage was not present. When all oesophageal tumours were considered in aggregate, the high number of squamous cell and signet ring tumours of advanced stage with altered E-cadherin expression led to a highly significant association between altered E-cadherin expression and advanced stage.

*Lymph node metastases* The most revealing tissue to examine for E-cadherin protein expression may be sites of metastatic disease. Local regional lymph nodes containing metastatic oesophageal cancer ($n = 12$), metastatic gastric cancer ($n = 3$) and metastatic lung cancer ($n = 2$) were examined immunohistochemically for E-cadherin protein expression (Figure 1h and l). All metastatic cells in lymph nodes exhibited intense E-cadherin expression at levels equal to and often greater than the primary tumour. None of the primary tumours of these patients exhibited preserved E-cadherin expression, six had reduced expression, nine were disorganised and two of the primary tumours were uninformative. While no metastatic sites exhibited reduced E-cadherin protein expression, 10 of 17 lymph nodes (59%) contained at least some metastatic cells which expressed E-cadherin in a disorganised fashion.

**Table I** E-cadherin protein expression patterns in oesophageal and lung tumours

| Stage | *E-cadherin pattern* Preserved | Reduced | Disorganised |
|---|---|---|---|
| *Oesophageal adenocarcinomas* ($n = 43$) | | | |
| I | 0 | 0 | 0 |
| IIa | 2 | 3 | 3 |
| IIb | 2 | 1 | 1 |
| III | 2 | 5 | 15 |
| IV | 0 | 6 | 3 |
| *Oesophageal squamous cell carcinomas* ($n = 8$) | | | |
| I | 0 | 0 | 0 |
| IIa | 1 | 0 | 0 |
| IIb | 0 | 0 | 0 |
| III | 0 | 2 | 3 |
| IV | 0 | 2 | 0 |
| *Other oesophageal carcinomas*[a] ($n = 8$) | | | |
| I | 0 | 0 | 0 |
| IIa | 0 | 0 | 0 |
| IIb | 0 | 1 | 1 |
| III | 0 | 5 | 1 |
| IV | 0 | 0 | 0 |
| *Lung adenocarcinomas* ($n = 52$) | | | |
| I | 9 | 6 | 14 |
| II | 1 | 1 | 4 |
| IIIa | 2 | 4 | 10 |
| IIIb | 0 | 0 | 0 |
| IV | 0 | 0 | 1 |

[a]Other contains four signet ring carcinomas, three gastric carcinomas of the gastro-oesophageal junction, one adenosquamous carcinoma.

**Table II** Statistical analysis of E-cadherin protein expression pattern and stage

| Stage | *E-cadherin pattern* Preserved | vs *altered*[a,b] | P-*value*[c] |
|---|---|---|---|
| *All oesophageal cancers* | | | |
| I | 0 | 0[d] | |
| IIa | 3 (1.1) | 6 (7.9) | |
| IIb | 2 (0.5) | 2 (3.5) | 0.008 |
| III | 2 (3.6) | 28 (26.4) | |
| IV | 0 (1.9) | 16 (14.1) | |
| *Oesophageal adenocarcinomas* | | | |
| I | 0 | 0[d] | |
| IIa | 2 (1.1) | 6 (6.9) | |
| IIb | 2 (0.6) | 2 (3.4) | 0.05 |
| III | 2 (3.1) | 20 (18.9) | |
| IV | 0 (1.3) | 9 (7.7) | |
| *Lung adenocarcinomas* | | | |
| I | 9 (6.7) | 20 (22.3) | |
| II | 1 (1.4) | 5 (4.6) | |
| IIIa | 2 (3.7) | 14 (12.3) | 0.55 |
| IIIb | 0 | 0[d] | |
| IV | 0 (0.2) | 1 (0.8) | |

[a]Altered consists of tumours with both reduced and disorganised expression. [b]Number of tumours observed and, in parenthesis, number of tumours expected given no association between E-cadherin protein expression pattern and stage. [c]Statistically significant association between altered E-cadherin protein expression and advanced stage based on Fisher's exact test (two-tail). [d]Stages in which no observations were made were not included in the analysis.

*Barrett's oesophagus* Twenty-nine specimens of Barrett's oesophagus were examined for E-cadherin protein expression (Figure 1c and d). Intestinal, junctional and fundic types of Barrett's metaplasia were associated with uniform, preserved staining of high intensity without exception. Fourteen dysplastic tissues exhibited a disorganised E-cadherin staining pattern with some areas exhibiting reduced staining (Figure 1d). Staining patterns varied even within the same gland, with dysplastic areas associated with altered E-cadherin expression and metaplastic areas exhibiting preserved expression.

*Northern blot analysis of E-cadherin and α-catenin*

Northern blot analysis was performed to examine the expression of E-cadherin mRNA in tumours and cell lines which express E-cadherin protein in either disorganised or reduced patterns. Lung adenocarcinomas with reduced E-cadherin protein expression were found to express an apparently intact E-cadherin mRNA at decreased but detectable levels (Figure 2a). Additionally, E-cadherin mRNA was nearly undetectable in the lung adenocarcinoma cell lines A549 and A427 (Figure 2b). These cell lines expressed essentially no E-cadherin protein, regardless of the state of growth and confluence of the cells in culture (not shown). Tumours with disorganised E-cadherin protein expression exhibited mRNA of correct size at variable levels equal to or greater than normal tissue (Figure 2a).

Tumours may retain preserved E-cadherin protein but might have altered α-catenin expression which could also contribute to defective cell adhesion and metastasis. Therefore, the expression of α-catenin mRNA was examined by Northern blot analysis in order to investigate this second possible defect leading to altered tumour cell adhesion (Figure 2b and c). Oesophageal cancers with preserved, reduced and disorganised E-cadherin protein expression and lymph nodes containing metastatic cancer were all found to express α-catenin mRNA. Interestingly, α-catenin mRNA is of correct size and expressed at high levels in both A549 and A427 cells (Figure 2b) and in tumours with reduced and disorganised E-cadherin expression (Figure 2c).

**Figure 2** **a**, Northern blot analysis of E-cadherin mRNA. Gastric tissue (G) and normal lung (L) are used as controls for high and low E-cadherin protein expressing tissues respectively. Lung adenocarcinomas expressing reduced E-cadherin protein are labelled T-red., while lung adenocarcinomas expressing disorganised E-cadherin protein are labelled T-disorg. 28S rRNA is used as a control for loading and transfer. **b**, Northern blot analysis of α-catenin mRNA. Normal stomach and lung (L) are used as controls. Lymph nodes containing metastatic oesophageal cancer (LN-met) and primary lung adenocarcinomas (T-dis, red, pres) express the same size mRNA transcripts as controls. 28S rRNA is used as a control for loading and transfer. **c**, Northern blot analysis of E-cadherin and α-catenin mRNA in A549 and A427 lung adenocarcinoma cells. E-cadherin protein is not detectable by immunohistochemistry in A549 and A427 cells. 28S rRNA is used as a control for loading and transfer.

### Discussion

E-cadherin has been hypothesised to represent a likely molecular target for altered cell adhesion in cancer. The present study demonstrates an association between altered E-cadherin protein expression and increased tumour stage, although the association did not reach statistical significance in the group of lung adenocarcinomas. Altered E-cadherin expression appears to exist in two distinct patterns, reduced staining and disorganised staining, although staining patterns are somewhat heterogeneous among tumours of each pattern and even within individual tumours. While the disorganised pattern occurred approximately two times more frequently than the reduced pattern, both patterns appear to identify tumours with altered cell adhesion with a similarly increased incidence of invasion and metastasis.

Examination of E-cadherin mRNA in tumours with various E-cadherin protein expression patterns suggests that there may be several possible mechanisms leading to altered E-cadherin protein expression in these cancers. Decreased levels of E-cadherin mRNA were seen in both primary tumours and lung adenocarcinoma cell lines with reduced protein expression (Figure 2a and b). This may be consistent with either a transcriptional down-regulation of the gene leading to reduced or absent E-cadherin protein or actual loss of the gene. In contrast, tumours with disorganised protein expression were found to have E-cadherin mRNA at similar levels and size as in normal tissue. This is consistent with the E-cadherin protein analysis, as there is often significant immunoreactivity in these tumours, although without the normal pattern that appears to be indicative of normal cell adhesion (Figure 1a,b and d). The disorganised protein expression present in thoracic neoplasms is potentially related to either post-transcriptional or post-translational events. An example of post-translational modification of cadherin is observed during embryonic development of the chick retina. The expression of N-cadherin, the form of cadherin molecule expressed in neural tissue, decreases in the developing chick retina as described by immunoblotting (Roark *et al.*, 1992). N-cadherin mRNA, however, remains constant over time, a situation analogous to that presented here in thoracic tumours with disorganised E-cadherin protein expression. Also, in medullary thyroid cancers E-cadherin mRNA levels remained constant while E-cadherin protein expression was variable (Brabant *et al.*, 1993). In the chick retina, the extracellular portion of the N-cadherin molecule is apparently cleaved by a metalloprotease (Roark *et al.*, 1992). Increased protease activity against E-cadherin or decreased protease inhibitor activity may account for the disorganised pattern of E-cadherin expression presented here. An altered E-cadherin protein, potentially modified by proteolysis, was detected by Western blot analysis in lung adenocarcinoma cells isolated from a malignant pleural effusion (Matsuura *et al.*, 1992). Similarly, Western blots for E-cadherin in Barrett's metaplasia and oesophageal adenocarcinoma revealed several bands of low molecular weight, possibly consistent with truncated protein or altered glycosylation (Jankowski *et al.*, 1994).

Strikingly, all 17 lymph nodes containing metastatic oesophageal, lung or gastric cancer examined in this study had high-level, preserved, E-cadherin protein expression. Furthermore, there were five tumours with preserved E-cadherin protein expression which were of advanced stage. The preserved pattern of E-cadherin expression in the metastatic cells seems inconsistent with an invasion/metastasis-suppressor role. Heterogeneity of tumours, with areas of preserved and reduced expression, could potentially lead to an inaccurate classification of some primary tumours. Cell–cell adhesion is a complex process involving multiple protein interactions, and other defects may exist in cases where tumour cells appear to invade or metastasise despite normal E-cadherin protein expression. A cytosolic protein associated with E-cadherin, α-catenin, has been shown to be critically important in normal cadherin function (Hirano *et al.*, 1992; Shimoyama *et al.*, 1992). Reduced expression of α-catenin protein was recently found to be correlated with invasion and metastases in squamous cell cancer of the oesophagus (Kadowaki *et al.*, 1994). A homozygous deletion of the α-catenin gene has been reported to occur in both a human bladder and lung cancer cell line leading to the loss of expression of α-catenin mRNA, protein and, significantly, a decrease in calcium-dependent cell aggregation (Hirano *et al.*, 1992; Morton *et al.*, 1993). In a fashion similar to transfections studies with E-cadherin described earlier (Frixen *et al.*, 1991; Velminckx *et al.*, 1991), transfection of α-catenin cDNA into PC9 lung adenocarcinoma cell results in restored aggregation properties (Hirano *et al.*, 1992). The α-catenin gene is apparently intact in the oesophageal adenocarcinoma metastases examined here, as abundant mRNA for α-catenin was detected. These metastatic cells also express E-cadherin protein in a preserved pattern (Figure 1h and l), which is known to be necessary for normal α-catenin protein expression (Nagafuchi *et al.*, 1991). Alterations in E-cadherin protein expression, however, may be transient, and affected by differing conditions present in the primary tumour and the metastatic site. The alteration might be at the level of E-cadherin transcription, translation or post-translational processing of the mature protein. A subset of cells within the primary tumour might transiently lose E-cadherin expression, metastasise and then re-express the protein at the distant site under different and perhaps E-cadherin-inducing conditions (i.e. lymph nodes). Alternatively, the observation that preserved E-cadherin protein expression is present at all metastatic sites examined may support the hypothesis that some degree of cell–cell adhesion is required for the formation of metastatic foci. Tumour cells which adhere as a mass of cells may have survival advantages both in circulation and at the metastatic site.

The association of altered E-cadherin expression with advanced stage in thoracic tumours is consistent with other reports implicating loss of E-cadherin as being an important factor in invasion and metastases in human malignancy. Additionally, altered expression of E-cadherin in Barrett's oesophagus provides important evidence that changes in cell adhesion may be early events in tumorigenesis. Preserved E-cadherin protein expression was observed in metaplastic Barrett's mucosa, however dysplastic Barrett's oesophagus demonstrated both reduced and disorganised E-cadherin protein expression patterns (Figure 1c and d). A similar finding of loss of E-cadherin expression in dysplastic Barrett's oesophagus as compared with normal squamous and metaplastic Barrett's oesophagus has recently been reported (Jankowski *et al.*, 1994). Altered E-cadherin protein expression in dysplastic Barrett's mucosa is probably associated with some degree of altered cell adhesion and importantly is present in tissues which may progress to adenocarcinoma but are not yet invasive. This finding is very much in accord with reported mutations within the *APC* gene present in premalignant, adenomatous polyps of the colon (Rubinfield *et al.*, 1993; Su *et al.*, 1993). The *APC* gene product is associated with β-catenin and is probably involved in cell adhesion. The interaction between E-cadherin and the actin cytoskeleton probably has direct affects upon cell morphology. Histological descriptions of dysplasia or the state of differentiation may be indirect descriptions of the state of cell adhesion.

### References

AL-KASSPOOLES M, MOORE JH, ORRINGER MB AND BEER DG. (1993). Amplification and overexpression of the EGFR and erbB2 genes in human esophageal adenocarcinomas. *Int. J. Cancer*, **54**, 1–7.

AMERICAN JOINT COMMITTEE ON CANCER STAGING. (1992). *Manual for Staging of Cancer*, 4th ed. JB Lippincott: Philadelphia.

BEHRENS J, MAREEL MM, VANROY FM AND BIRCHMEIER W. (1989). Dissecting tumor cell invasion: epithelial cells acquire invasive properties after loss of uvomorulin-mediated cell–cell adhesion. *J. Cell Biol.*, **108**, 2435–2447.

BOWIE GL, CASLIN AW, ROLAND NJ, FIELD JK, JONAS AS AND KINSELLA AR. (1993). Expression of cell–cell adhesion molecule E-cadherin in squamous cell carcinoma of the head and neck. *Clin. Otolaryngol.*, **18**(3), 196–201.

BRABANT G, HOANG-VU C, CETIN Y, DRALLE H, SCHEUMANN G, MOLNE J, HANSSON G, JANSSON S, ERICSON E AND NILSSON M. (1993). E-cadherin: a differentiation marker in thyroid malignancies. *Cancer Res.*, **53**, 4987–4993.

BRINGUIER PP, UMBAS R, SCHAAFSMA HE, KARTHAUS HFM, DEBRUYNE FMJ AND SCHALKEN JA. (1993). Decreased E-cadherin immunoreactivity correlates with poor survival in patients with bladder tumors. *Cancer Res.*, **53**, 3241–3245.

BUSSMAKERS MJ, VAN BOKHOVEN A, MEES SG, KEMLER R AND SCHALEN JA. (1993). Molecular cloning and characterization of the human E-cadherin cDNA. *Mol. Biol. Rep.*, **17**, 123–128.

DORUDI S, SHEFFIELD JP, POULSOM R, NORTHOVER JMA AND HART IR. (1993). E-cadherin expression in colorectal cancer. *Am. J. Pathol.*, **142**(4), 981–986.

EIDELMAN S, DAMSKY CH, WHEELOCK MJ AND DAMJANOV I. (1989). Expression of the cell-cell adhesion glycoprotein cell-CAM120/80 in normal human tissues and tumors. *Am. J. Pathol.*, **135**, 101–110.

FRIXEN UH, BEHRENS J, SACHS M, EBERKE G, VOSS B, WARDA A, LOCHNER D AND BIRCHMEIER W. (1991). E-cadherin-mediated cell-cell adhesion prevents invasiveness of human carcinoma cells. *J. cell Biol.*, **112**, 173–185.

HANSON LA, NUZUM EO, JONES BC, MALKINSON AM AND BEER DG. (1991). Expression of the glucocorticoid receptor and k-*ras* genes in urethane induced mouse lung tumors and transformed cell lines. *Exp. Lung Res.*, **17**, 371–387.

HIRANO S, KIMOTO N, SHIMOYAMA Y, HIROHASHI S AND TAKEICHI M. (1992). Identification of a neural α-catenin as a key regulator of cadherin function and multicellular organization. *Cell*, **70**, 293–301.

JANKOWSKI J, NEWHAM P, NEWHAM P, KANDEMIR O, HIRANO S, TAKEICHI M AND PIGNATELLI M. (1994). Differential expression of E-cadherin in normal, metaplastic and dysplastic oesophageal mucosa: a putative biomarker. *Int. J. Oncol.*, **4**, 441–448.

KADOWAKI T, SHIOZAKI H, INOUE M, TAMURA S, OKA H, DOKI Y, IIHARA K, MATSUI S, IWAZAWA T, NAGAFUCHI A, TSUKITA S AND MORI T. (1994). E-cadherin and α-catenin expression in human esophageal cancer. *Cancer Res.*, **54**, 291–296.

KEMLER R. (1993). From cadherins to catenins: cytoplasmic protein interactions and regulation of cell adhesion. *Trends Genet.*, **9**, (90) 317–321.

KINSELLA AR, GREEN B, LEPTS GC, HILL CL, BOWIE G AND TAYLOR BA. (1992). The role of the cell-cell adhesion molecule E-cadherin in large bowel tumour cell invasion and metastasis. *Br. J. Cancer*, **67**, (5) 804–809.

LEE SW, TOMASETTO C, PAUL D, KEYOMARSI K AND SAGER R. (1992). Transcriptional down regulation of gap junction proteins blocks junctional communication in human mammary tumor cell lines. *J. Cell Biol.*, **118**, 1213–1221.

MAREEL MM, BEHRENS J, BIRCHMEIER W, DEBRUYNE GK, VLEMINCKX K, HOOGEWUS A, FIERS WC AND VANROY FM. (1991). Down-regulation of E-cadherin expression in Madin-Darby canine kidney (MDCK) cells inside tumors of nude mice. *Int. J. Cancer*, **47**, 922-928.

MATSUURA K, KAWANISHI J, FUJII S, IMAMURA M, HIRANO S, TAKEICHI M AND NIITSU Y. (1992). Altered expression of E-cadherin in gastric tissues and carcinomatous fluid. *Br. J. Cancer*, **66**, 1122-1130.

MAYER B, JOHNSON JP, LEITL F, JAUCH KW, HEISS MM, SCHILDBERG FW, BIRCHMEIER W AND FUNKE I. (1993). E-cadherin expression in primary and metastatic gastric cancer: down-regulation correlates with cellular dedifferentiation and glandular disintegration. *Cancer Res.*, **53**, 1690-1695.

MEHTA CR AND PATEL NR. (1983). A network algorithm for performing Fisher's exact test in $r \times c$ contigency tables. *J. Am. Stat. Assoc.*, **78**, 427-434.

MORTON RA, EWING CM, NAGAFUCHI A, TSUKITA S AND ISAACS WB. (1993). Reduction of E-cadherin levels and deletion of the α-catenin gene in human prostate cancer cells. *Cancer Res.*, **53**, 3585-3590.

NAGAFUCHI A, SHIRAYOCHI Y, OKAZAKI K, YASUDA K AND TAKEICHI M. (1987). Transformation of cell adhesion properties by exogenously introduced E-cadherin cDNA. *Nature*, **329**, 341-343.

NAGAFUCHI A, TAKECHI M AND TSUKITA S. (1991). The 102 kD cadherin-associated protein: similarity to vinculin and post-transcriptional regulation of expression. *Cell*, **65**, 849-857.

ODA T, KANAI Y, SHIMOYAMA Y, NAGAFUCHI A, TSUKITA S AND HIROHASHI S. (1993). Cloning of the human α-catenin cDNA and its aberrant mRNA in a human cancer cell line. *Biochem. Biophys. Res. Commun.*, **193**, 897-904.

OKA H, SHIOZAKI H, KOBAYASHI K, INOUE M, TAHARA H, KOBAYASHI T, TAKATSUKA Y, MATSUYOSHI N, HIRANO S, TAKEICHI M AND MORI T. (1993). Expression of E-cadherin cell adhesion molecule in human breast cancer tissues and its relationship to metastasis. *Cancer Res.*, **53**, 1696-1701.

PIEPENHAGEN PA AND NELSON WJ. (1993). Defining E-cadherin-associated protein complexes in epithelial cells: plakoglobin, β- and γ-catenin are distinct components. *J. Cell Sci.*, **104**, 751-762.

ROARK EF, PARADIES NE, LAGUNOWICH LA AND GRUNWALD GB. (1992). Evidence for endogenous proteases, mRNA levels and insulin as multiple mechanisms of N-cadherin down-regulation during retinal development. *Development*, **114**, 973-984.

RUBINFIELD B, SOUZA B, ALBERT I, MULLER O, CHAMBERLAIN SH, MASIARZ S, MUNEMITSU S AND POLAKIS P. (1993). Association of the APC gene product with β-catenin. *Science*, **262**, 1731-1734.

SANGER F, NICKLEN S AND COULSON AR. (1977). DNA sequencing with chain terminating inhibitors. *Proc. Natl Acad. Sci. USA*, **74**, 5463-5467.

SHIMOYAMA Y, HIROHASHI S, HIRANO S, NOGUCHI M, SHIMOSATO Y, TAKEICHI M AND ABE O. (1989). Cadherin cell-adhesion molecules in human epithelial tissue and carcinomas. *Cancer Res.*, **49**, 2128-2133.

SHIMOYAMA Y, NAGAFUCHI A, FUJITA S, GOTOH M, TAKEICHI M, TSUKITA S AND HIROHASHI S. (1992). Cadherin dysfunction in a human cancer cell line: possible involvement of loss of α-catenin expression in reduced cell-cell adhesiveness. *Cancer Res.*, **52**, 5770-5774.

SU L-K, VOGELSTEIN B AND KINZLER KW. (1993). Association of the APC tumor suppressor protein with catenins. *Science*, **262**, 1734-1736.

TAKEICHI M. (1990). Cadherins: a molecular family important in selective cell-cell adhesion. *Annu. Rev. Biochem.*, **59**, 237-252.

TAKEICHI M. (1991). Cadherin cell adhesion receptors as a morphogenetic regulator. *Science*, **251**, 1451-1455.

TAKEICHI M. (1993). Cadherin in cancer: implications for invasion and metastasis. *Curr. Opin. Cell Biol.*, **5**, 806-811.

UMBAS R, SCHALKEN JA, AALDERS TW, CARTER BS, KARTHAUS HFM, SCHAAFSMA HE, DEBRUYNE FMJ AND ISSACA WB. (1992). Expression of the cellular adhesion molecule E-cadherin is reduced or absent in high-grade prostate cancer. *Cancer Res.*, **52**, 5104-5109.

VLEMINCKX K, VAKAET Jr. L, MAREEL M, FIERS W AND VANROY F. (1991). Genetic manipulation of E-cadherin expression by epithelial cells reveals an invasion suppressor role. *Cell*, **66**, 107-119.